# A typology of cerebral small vessel disease based on imaging markers

**DOI:** 10.1007/s00415-023-11831-x

**Published:** 2023-06-27

**Authors:** Christoph Sperber, Arsany Hakim, Laura Gallucci, David Seiffge, Beata Rezny-Kasprzak, Eugen Jäger, Thomas Meinel, Roland Wiest, Urs Fischer, Marcel Arnold, Roza Umarova

**Affiliations:** 1 Department of Neurology, Inselspital, University Hospital Bern, University of Bern, Bern, Switzerland; 2 University Institute of Diagnostic and Interventional Neuroradiology, Inselspital, University Hospital Bern, University of Bern, Bern, Switzerland; 3 Department of Neurology, University Hospital Basel, University of Basel, Basel, Switzerland

**Keywords:** White matter hyperintensities, Lacune, Microbleed, Enlarged perivascular spaces, Stroke outcome, k-means

## Abstract

**Background:**

Lacunes, microbleeds, enlarged perivascular spaces (EPVS), and white matter hyperintensities (WMH) are brain imaging features of cerebral small vessel disease (SVD). Based on these imaging markers, we aimed to identify subtypes of SVD and to evaluate the validity of these markers as part of clinical ratings and as biomarkers for stroke outcome.

**Methods:**

In a cross-sectional study, we examined 1207 first-ever anterior circulation ischemic stroke patients (mean age 69.1 ± 15.4 years; mean NIHSS 5.3 ± 6.8). On acute stroke MRI, we assessed the numbers of lacunes and microbleeds and rated EPVS and deep and periventricular WMH. We used unsupervised learning to cluster patients based on these variables.

**Results:**

We identified five clusters, of which the last three appeared to represent distinct late stages of SVD. The two largest clusters had no to only mild or moderate WMH and EPVS, respectively, and favorable stroke outcome. The third cluster was characterized by the largest number of lacunes and a likewise favorable outcome. The fourth cluster had the highest age, most pronounced WMH, and poor outcome. Showing the worst outcome, the fifth cluster presented pronounced microbleeds and the most severe SVD burden.

**Conclusion:**

The study confirmed the existence of different SVD types with different relationships to stroke outcome. EPVS and WMH were identified as imaging features of presumably early progression. The number of microbleeds and WMH severity appear to be promising biomarkers for distinguishing clinical subgroups. Further understanding of SVD progression might require consideration of refined SVD features, e.g., for EPVS and type of lacunes.

**Supplementary Information:**

The online version contains supplementary material available at 10.1007/s00415-023-11831-x.

## Introduction

Cerebral small vessel disease (SVD) has been identified to cause numerous neurological and neuropsychiatric pathologies, including stroke [1], dementia [2, 3], depression [4], and motor disability [5]. SVD is a disease of the cerebral arterioles, capillaries, and venules [6] and, in its later stages, it is linked to abnormalities visible on MRI. These include white matter hyperintensities (WMH), lacunes, enlarged perivascular spaces (EPVS), and microbleeds [7–9]. The consideration of several or all of these imaging features together might improve the prediction of conversion to neurological and neuropsychiatric pathologies [6] and prognostic indices incorporating several or all of these features were generated [7, 10–12].

The generation and optimization of such indices bear several challenges. Some of the imaging features are common in cognitively unaffected elderlies, they only indirectly indicate pathology of the vessels, and their clinical significance and cutoffs to define relevant pathology are unclear [13]. Moreover, such an index is blind to possible heterogeneous disease trajectories that could lead to different subgroups of patients. Still, such SVD indices, including simple scoring that only counts the presence of the four imaging features [7, 8] were successfully used as biomarkers for cognitive status, dementia, post-stroke outcome or depression [13]. Given the marked complexity of SVD and other pathological processes that potentially interfere with the emergence of lesions, a differentiation of potential subgroups of SVD might be clinically informative and allow better characterization in derived disease biomarkers.

In the present study, we investigated the typology of imaging features for SVD by unsupervised learning algorithms in a cross-sectional sample of stroke patients. The clinical relevance of our findings was evaluated by reference to stroke outcome, which previous studies found to be associated with SVD [14–16].

## Methods

### Subjects and clinical assessment

**Fig. 1:**
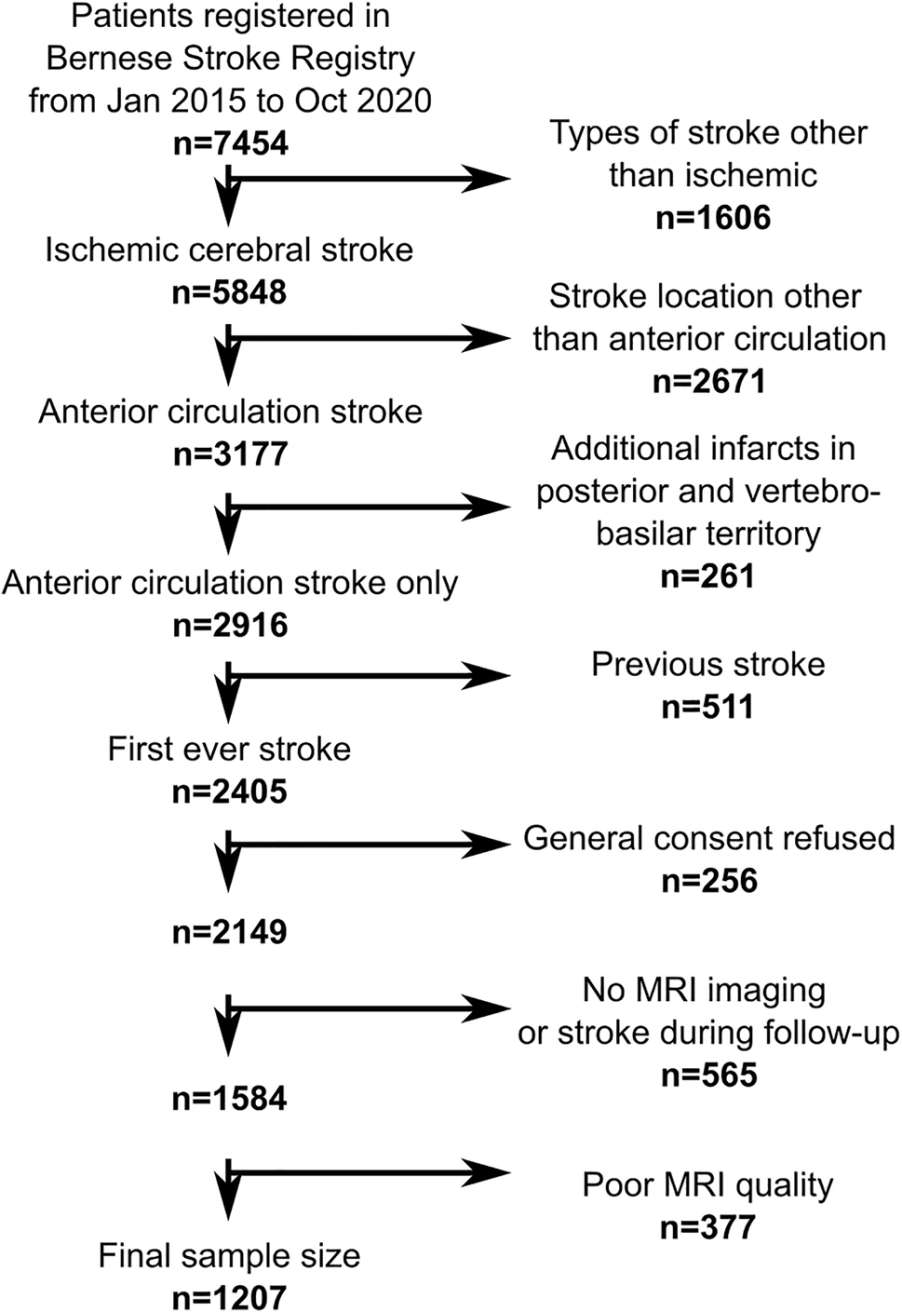
Patient flowchart

This retrospective cohort study included patients from the Bern Stroke Registry admitted to the tertiary Bern Stroke Center for treatment of acute ischemic stroke between January 2015 and October 2020. The catchment area is an urban center with rural surroundings in Switzerland with a predominantly European Caucasian population. Inclusion criteria were: (1) a first-ever ischemic anterior circulation stroke confirmed in admission MRI examination, (2) available MRI examination, which has been acquired routinely either at admission or 24 h post-stroke, and (3) general consent for scientific data use. We excluded patients who (1) suffered an additional stroke in the posterior circulation to avoid heterogeneity introduced by distinct functional recovery trajectories in posterior circulation stroke or (2) with poor MRI quality. Furthermore, because this study was part of an overarching project focused on clinical outcomes, we excluded patients that suffered from (3) a recurrent stroke during follow-up as it changes substantially the recovery trajectories. The presence or absence of any imaging features of SVD was not considered in the inclusion criteria, and patients without any visible MRI features of SVD were included. Stroke etiology was also not considered in the inclusion criteria. The rate of small vessel occlusion as stroke etiology was comparatively low (Table 1), which may be explained by the broad etiologic workup that often attributed stroke to causes other than SVD even in small lacunar strokes. In line with this interpretation, the rate of patients with small vessel occlusion was the same in the excluded patients. Figure 1 shows a recruitment flow chart. All patients underwent standardized clinical assessment and treatment according to the Bern Stroke Guidelines and standardized rehabilitation after discharge. Written informed consent was available from all participants or their guardians. The study was performed in line with the Declaration of Helsinki and approval from the local ethical committee (Kantonale Ethikkommission Bern KEK) was received.

**Table 1:** Demographics and clinical data

All participants, *N* = 1207	
Age, years mean (SD; range)	69.1 (15.4; 16–98)
Sex, male %	56.8
History of transient ischemic attack, %	5.7
Hypertension, %	70.6
Diabetes, %	17.3
Smoking, %	26.2
Hyperlipidemia, %	68.3
Atrial fibrillation, %	27.5
Coronary heart disease, %	16.9
Body mass index, mean (SD)	26.4 (4.7)
Pre-stroke mRS, median [quartiles]	1 [1, 3]
3 months mRS, median [quartiles]	0 [0, 1]
NIHSS 24 h, mean (SD)	5.3 (6.8)
Stroke etiology (extended TOAST criteria)	
Cardiac embolism, no./%	362/30.0%
Cervical artery dissection, no./%	48/4.0%
Large artery atherosclerosis, no./%	175/14.5%
Patent foramen ovale, no./%	65/5.4%
Small vessel occlusion, no./%	40/3.3%
More than one possible etiology, no./%	81/6.7%
Other determined etiology, no./%	47/3.9%
Unknown despite complete evaluation, no./%	230/19.1%
Unknown with incomplete evaluation, no./%	158/13.1%
SVD measures	
Lacunes present, no./%	290/24.0%
No. lacunes when present, median [quart.]	1 [1; 3]
Microbleeds present, no./%	315/26.1%
No. Microbl. when present, median [quart.]	2 [1; 3]
EPVS, median [quartiles]	2 [1; 3]
Periventricular WMH, median [quartiles]	1 [0; 2]
Deep WMH, median [quartiles]	1 [0; 1]
SVD MRI burden, median [quartiles]	1 [0; 2]

Note that some demographic or clinical variables contained missing values (not more than 12.1% of the total sample) which were omitted in the computation of characteristics

*mRS* modified Rankin Scale, *SD* standard deviation, *NIHSS* National Institutes of Health Stroke Scale, *SVD* small vessel disease, *WMH* white matter hyperintensities

At admission, patients were assessed for pre-stroke functional disability (pre-stroke modified Rankin Scale, mRS) and stroke severity using the National Institutes of Health Stroke Scale (NIHSS) by a certified neurologist. Cardiovascular risk factors were acquired from the medical records or patient interviews. Stroke outcome was assessed as functional disability (mRS) 3 months post-stroke.

### Brain MRI acquisition

We analyzed clinical routine MRI acquired at admission or within the first 24 h post-stroke onset. Scans were obtained with a 1.5 T or 3 T scanner (Magnetom Avanto, Aera, Skyra, Verio, Vida, Siemens Medical Solutions, Erlangen, Germany). The assessment of SVD lesions required a set of MRI sequences that included axial diffusion-weighted imaging (DWI) with apparent diffusion coefficient (4–5 mm slice thickness), axial fluid-attenuated inversion recovery (FLAIR; 4–5 mm slice thickness), axial susceptibility-weighted imaging (1.5 mm slice thickness) and axial T1-weighted imaging (4–5 mm slice thickness).

### Assessment of small vessel disease

The four major imaging features of SVD composing the SVD score were rated: WMH pronouncement, the total number of lacunes, the total number of microbleeds, and the number of EPVS. We did not evaluate the SVD features of cerebral amyloid angiopathy such as cortical superficial siderosis as our focus was hypertensive/age-related SVD, due to the low probability of cerebral amyloid angiopathy in ischemic stroke populations and potential confounders such as previous head trauma etc. The numbers of all lacunes and microbleeds were counted across the whole brain. Lacunes were defined as rounded or ovoid cavities of > 3 and < 20 mm with cerebrospinal fluid intensity and surrounding gliosis on FLAIR imaging [8, 17]. Microbleeds were defined as small (< 5 mm) homogeneous hypointensities in the susceptibility-weighted imaging scan and were differentiated from mineralization or calcification of the globus pallidus, suspected cavernoma with popcorn pattern, and petechial hemorrhage within the infarct [18]. Enlarged perivascular spaces (EPVS) were defined as small punctuate or linear lesions in the basal ganglia with cerebrospinal fluid equivalent signal and were assessed on a previously validated semiquantitative scale (range 0–4) in the hemisphere with more visible pathology on the level of basal ganglia with more pronounced changes [19]. Note that EPVS are commonly assessed with a T2 sequence. As this was not included in the local acute clinical protocol, the neuroradiologists performed the rating based on the B0 DWI image and ADC map in comparison with the FLAIR and T1 images. White matter hyperintensities (WMH) were defined as hyperintense signal abnormalities in FLAIR imaging surrounding the ventricles or in the deep white matter and were rated with the Fazekas scale [20] separately for periventricular and deep WMH (range 0–3).

Each type of SVD lesion was additionally assessed by a binary rating derived from the continuous or ordinal rating variables described above. The binarization followed a rating scheme for the total MRI burden of SVD [7, 8]. The variables included (1) the presence of lacunes (lacunes ≥ 1), (2) the presence of microbleeds (microbleeds ≥ 1), and (3) the presence of at least a moderate amount of EPVS in the basal ganglia (ordinal rating ≥ 3) [19]. (4) We noted the presence of pathologically relevant WMH if the rating indicated a beginning or already existing large confluence of hyperintense loci in the deep white matter (deep white matter Fazekas score ≥ 2) or/and irregular hyperintensities in the periventricular white matter that extending into the deep white matter (periventricular Fazekas score = 3) [20]. A score for total SVD MRI burden [7, 8] was computed by summation of the binary evaluations for lacunes, microbleeds, WMH, and EPVS, resulting in a scale of 0–4. Following the cluster analysis and considering the different prevalences of relevant deep and periventricular WMH, additional binary variables were created separately for each of the two types of WMH with the same cutoffs for the estimation of disease progression models. SVD raters were blinded to further clinical information.

### Data modeling and statistical analysis

We clustered the patients into groups according to their SVD feature profile by *k*-means clustering in MATLAB with 1000 replications. This algorithm classifies the patients so that resulting groups are as similar as possible in regards to their SVD pathology. We entered the quantitative data for lacunes, microbleeds, EPVS, periventricular WMH and deep WMH into the analysis. The numbers of lacunes and microbleeds were found to be skewed and were log-transformed, and all variables were normalized. We determined the optimal number of clusters *k* by comparison of the Silhouette and Davies–Bouldin criteria. We compared relevant clinical and demographic variables between the clusters with one-way ANOVAs or Kruskal–Wallis tests with HSD-corrected post hoc comparisons. To include the collection of risk factors within a concise statistical analysis, we summed the presence of diabetes, hypertension, smoking, hyperlipidemia, atrial fibrillation, and coronary heart disease into a risk factor index between 0 and 6. Some variables contained missing values, which reduced the total sample size for these sub-analyses by between 0 and 12.1%.

In a second analysis that aimed to infer the typical disease progression of SVD markers, we modeled the progression of the different types of SVD-related lesions with Bayesian tree models that are capable to represent the progression of binary disease events from cross-sectional data [21]. This analysis failed to identify a complete progression structure within the data and did not create improved stroke outcome biomarkers. Due to these null results and the journal’s word limitation, we report the methods and results of this analysis only in the supplementary.

## Results

We included 1207 first-ever stroke patients in this retrospective study. Table 1 shows demographic and clinical data. All SVD features correlated weakly with each other (0.07 ≤ *r* ≤ 0.27; Table 2), except for the two different ratings for deep and periventricular WMH, which correlated highly (*r* = 0.79; *p* < 0.001). The correlation between age and SVD features varied from weak or non-existent for lacunes and microbleeds to medium for EPVS or deep WMH to strong (*r* = 0.51; *p* < 0.001) for periventricular WMH (Table 2). The correlation between mRS at 3 months and SVD features varied from none for enlarged perivascular spaces to very weak for the number of lacunes to weak for the number of microbleeds and WMH. The highest correlation was found for deep and periventricular WMH (both *r* = 0.21; *p* < 0.001).

**Table 2:** Correlations between SVD measures and demographic and clinical variables

	Lacunes	Microbleeds	EPVS	WMH PV	WMH D
Lacunes	1	0.13/0.15	0.12/0.10	0.19/0.16	0.19/0.18
Microbleeds		1	0.07/0.12	0.11/0.20	0.11/0.19
1EPVS			1	0.27/0.23	0.26/0.24
WMH PV				1	0.79/0.71
WMH D					1

	Age	Pre-stroke mRS	Post-stroke mRS	NIHSS 24 h
Lacunes	0.10/0.12	0.07/0.08	0.08/0.08	*0.01*/*0.02*
Microbleeds	*0.01*/0.13	*0.00*/0.05	0.15/0.14	*0.01*/0.08
EPVS	0.31/0.22	0.07/*0.05*	*0.02*/*0.02*	*0.00*/*0.04*
WMH PV	0.52/0.41	0.23/0.21	0.21/0.16	0.12/0.13
WMH D	0.46/0.37	0.19/0.16	0.21/0.17	0.13/0.14
SVD burden	0.40/0.32	0.16/0.13	0.19/0.14	0.07/0.11

Pearson’s correlation *r* and Kendall’s rank correlation *τ* between SVD features and demographic variables are shown. Correlations were computed based on partially skewed, non-transformed values, which is the reason for reporting both correlation measures. In the upper table, all correlations were significant at *p* < 0.05. In the lower table, non-significant correlations are shown in italic

*EPVS* enlarged perivascular spaces, *WMH PV* periventricular white matter hyperintensities, *WMH D* deep white matter hyperintensities, *mRS* modified Rankin Scale

### Cluster analysis

**Fig. 2:**
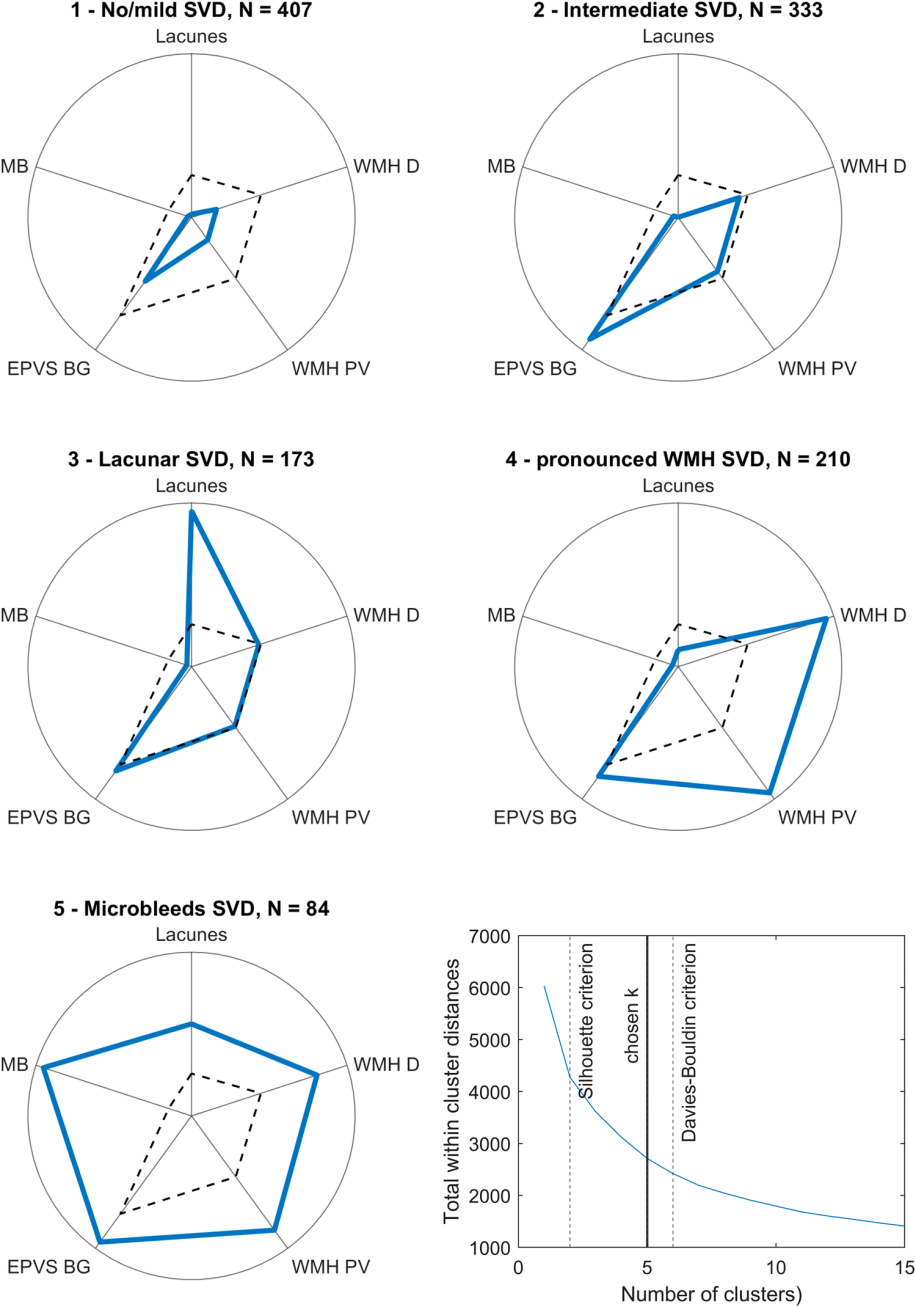
Clusters identified by k-means clustering on SVD MRI features. Comparison of SVD features profiles across clusters. For intuitive visualization, each variable was re-transformed into the original data space and has been scaled to the highest mean in any cluster. Detailed scores with mean and standard deviation are shown in Supplementary table 1. Dashed black lines indicate the mean score across the total sample. The last panel plots the total within-cluster distances across *k*-means cluster solutions for 1 ≤ *k* ≤ 15. *MB* microbleeds, *EPVS BG* enlarged perivascular spaces in the basal ganglia, *WMH PV* periventricular white matter hyperintensities, *WMH D* deep white matter hyperintensities

The optimal number of clusters *k* to represent the data was estimated with 2 by the Silhouette criterion and 6 by the Davies–Bouldin criterion. We consulted a plot of the total within-cluster distances (Fig. 2) to find an optimal number within this range. No single ‘elbow’ point became apparent, but a corresponding curvature at 4 ≤ *k* ≤ 6, and therefore picked *k* = 5 as the number of clusters.

**Fig. 3:**
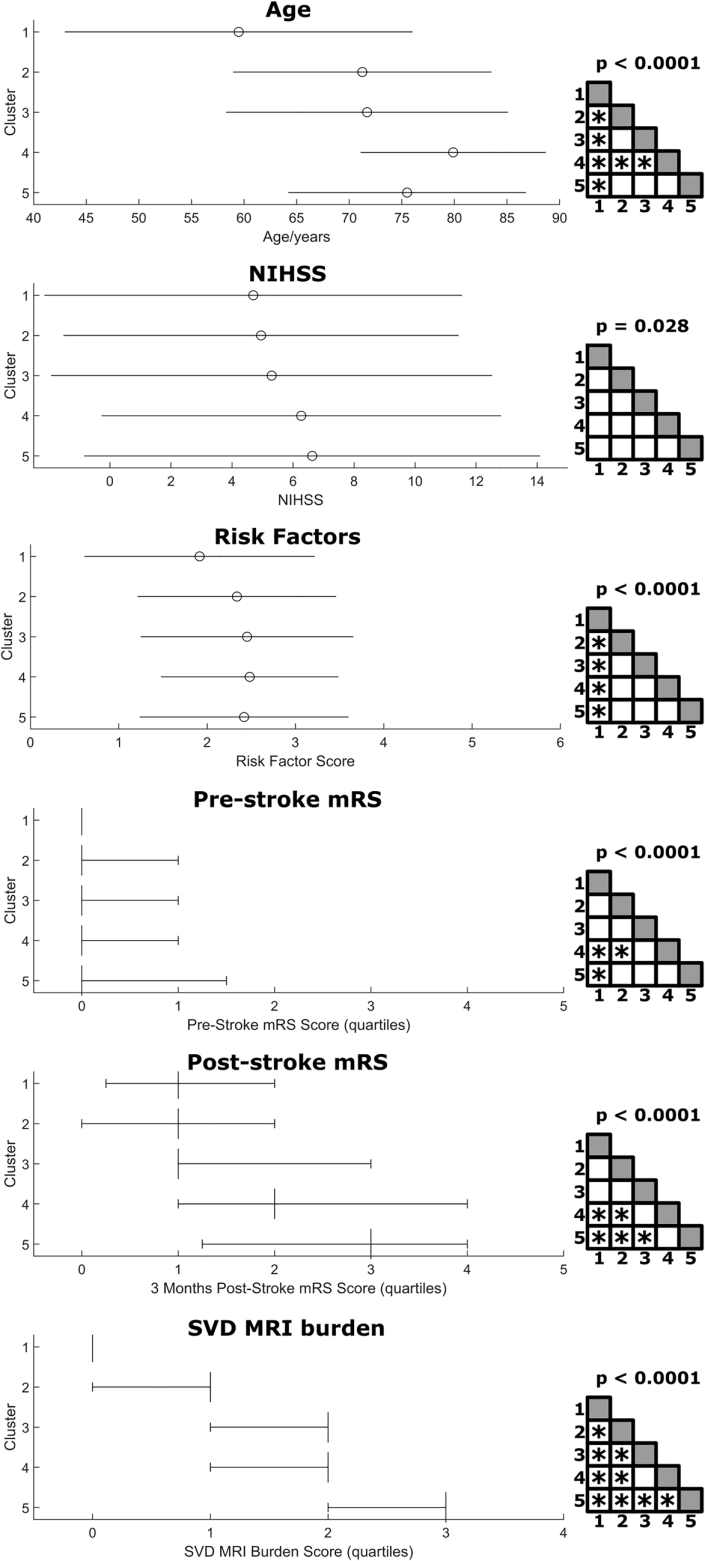
Comparison of clinical and demographic variables across clusters. Statistical comparison of demographic and clinical variables across the five clusters. The upper three plots show mean + standard deviation, the lower three plots median + quartiles. The elements on the right show the significance of the overall statistical test (one-way ANOVA or Kruskal–Wallis test) and the results of HSD-corrected post hoc tests; asterisks indicate a significant post hoc test at a corrected *p* < 0.05. Detailed statistics are shown in Supplementary table 2

The resulting solution clustered the sample into groups of 84–407 patients (Fig. 2). Comparison of demographic and clinical variables between clusters (Fig. 3), including age, stroke severity (NIHSS 24 h after stroke), risk factor score, pre-stroke functional disability (pre-stroke mRS) and stroke outcome (mRS 3 months post-stroke), and SVD MRI burden score found significant differences in omnibus tests for all variables (Supplementary Table 2). Notably, post hoc tests did not indicate any group differences for NIHSS 24 h meaning that stroke severity was comparable between clusters. The SVD MRI burden score significantly differed between all clusters except clusters 3 and 4. The risk factor rating only differed between cluster 1 and all other clusters, but not between clusters 2 and 5.

Cluster 1 (no/mild SVD) was the largest cluster (*N* = 407) and exhibited the lowest SVD scores across all variables, which strongly suggests that it represented patients that did have no or only mild SVD. In line with this interpretation, the patients in this cluster were the youngest (mean age = 59.5 years), their risk factor score was the lowest, and pre-and post-stroke mRS were numerically the lowest and significantly lower than in clusters 4 and 5. Given this interpretation, SVD-related abnormalities were assessed in relation to this cluster.

Cluster 2 (intermediate SVD) was the second largest cluster (*N* = 333) and was characterized by median WMH, above-average EPVS, and little to no lacunes or microbleeds. Although the SVD MRI burden score was significantly larger than in the first cluster, neither pre- nor post-stroke mRS significantly differed.

Cluster 3 (lacunar SVD; *N* = 173) was characterized by the highest number of lacunes among all clusters and the presence of at least one lacune in each patient (mean *n* = 2.1), whereas WMH and EPVS were average, and still little to no microbleeds were present. And even though this cluster had a significantly higher SVD MRI burden score than the two previous clusters, neither pre- nor post-stroke mRS significantly differed from them, i.e., stroke outcome was not worsened.

Cluster 4 (pronounced WMH SVD; *N* = 210) was characterized by the most pronounced WMH and above-average enlarged perivascular spaces. On the other hand, lacunes and microbleeds were relatively rare. The most striking feature was the high age (mean = 79.9 years), which was numerically the largest and significantly larger than in the first three clusters. The pronounced WMH together with the high age was in line with the medium to strong correlations between both variables (*r* = 0.46/0.52; see Table 2). This cluster’s SVD MRI burden score was comparable to cluster 3, but, contrary to cluster 3, patients in this cluster suffered from significantly higher pre- and post-stroke mRS than patients in cluster 1.

Cluster 5 (microbleeds SVD; *N* = 84) was characterized by pronounced pathology in all SVD variables, whereas the most striking feature was the strong presence of microbleeds. All patients suffered from at least one microbleed (mean *n* = 10.7), while they were comparatively rare in the other clusters (mean 0.28 < *n* < 0.36). Lacunes were common (mean *n* = 1.7) and present in 55% of all patients; WMH and EPVS were severe. In line with the marked brain pathology, the SVD MRI burden score was highest in this cluster, as was pre- and post-stroke mRS, both of which were significantly higher compared with the first cluster.

**Fig. 4:**
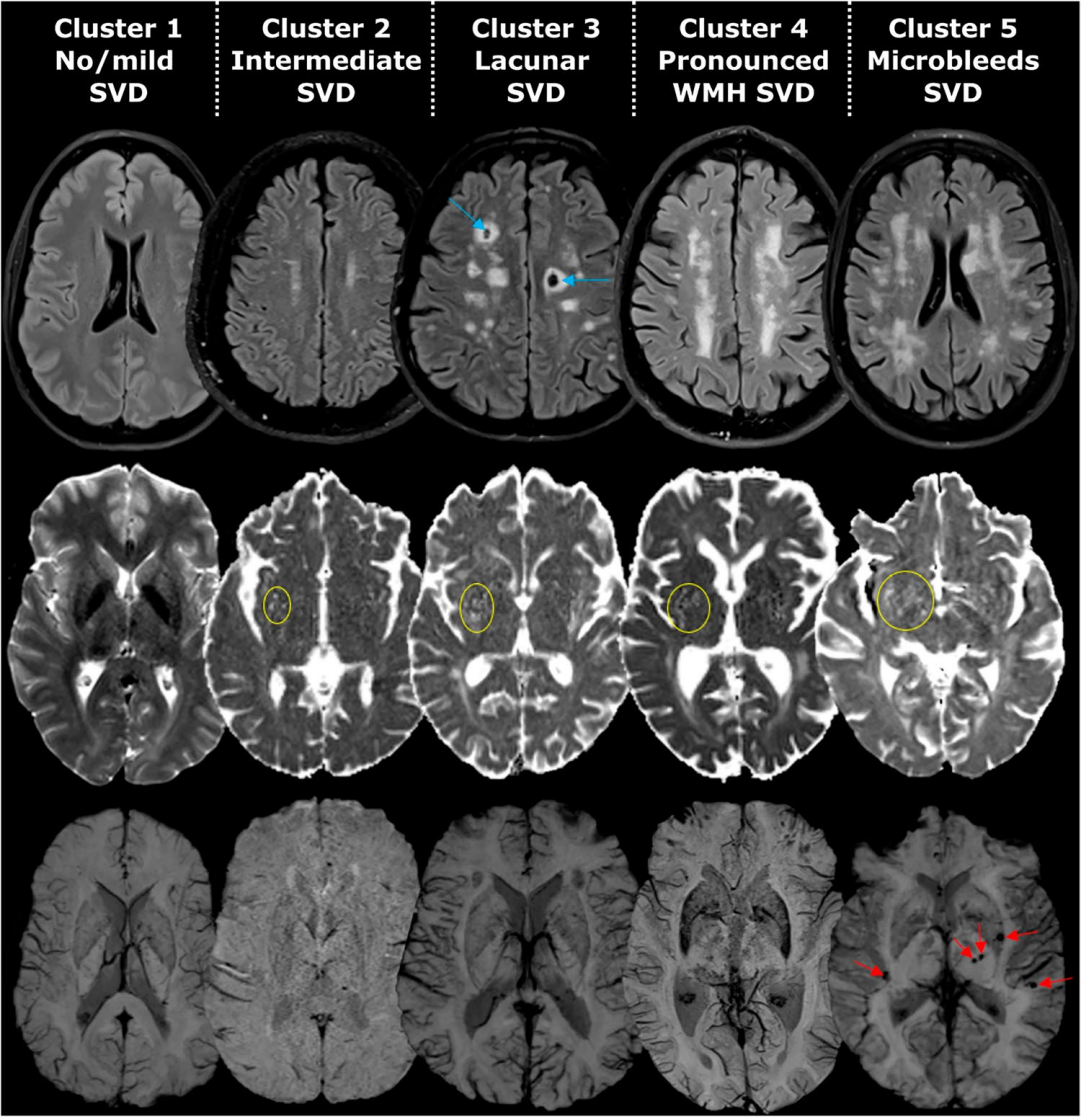
Example MRIs for each cluster. T2 FLAIR, DWI b0, and susceptibility-weighted MRI of example patients in each cluster. Coloured markings highlight the relevant SVD features (blue lacunes, yellow EPVS, red microbleeds)

Figure 4 shows example MRIs for patients in each cluster. In a control analysis reported in the supplementary, we investigated if MRI field strength could have influenced the current results. In short, some SVD features were significantly higher with a 3 T MRI scanner. However, underestimation of SVD features by 1.5 T MRI scanner did not affect the results, as a re-analysis of only patients assessed with a 3 T scanner created conceptually the same clusters as the analysis in the total sample.

## Discussion

We clustered patients into five groups based on imaging features of SVD. The cluster profiles of SVD features and the differences in clinical and demographic variables suggest different processes and stages underlying the development of the pathology visible in MRI.

The first cluster (no/mild SVD) was only minimally or not affected by visible SVD features, representing a comparably young reference group that is presumably (yet) unaffected by SVD. The second cluster (intermediate SVD) with moderate WMH and EPVS appeared to be a potential intermediate pathology stage and would allow progression to any of the remaining clusters. The disease progression models reported in the supplementary estimated WMH and EPVS as typical early disease features and thereby strengthened this interpretation. The third cluster (lacunar SVD) differed by pronounced lacunes, but still with a favorable outcome. The last two clusters were associated with poor stroke outcome. The fourth cluster (pronounced WMH SVD) was characterized by the most pronounced WMH and oldest age. The fifth cluster (microbleeds SVD) showed the largest overall burden of SVD features, including microbleeds. This feature differentiated this cluster from the others. While the SVD feature profiles alone might suggest that the last two clusters are successive late stages of the disease, the higher age of the fourth cluster argues against such an interpretation. Instead, two distinct processes behind clusters 4 and 5 appear more likely. While the fifth cluster (microbleeds SVD) presents all expected features of late-stage SVD, the fourth cluster (pronounced WMH SVD) might represent pathological aging effects. In line with the point, WMH, but not microbleeds or lacunes were demonstrated to be associated with frailty [22] and transition to disability [23] in elderlies. Overall, our findings suggest different types of SVD with a distinct impact on stroke outcome. To our best knowledge, neuropathological studies that distinguished different SVD types are missing until now [24], and future studies are needed to close this gap.

The presence and number of lacunes and microbleeds appear to be features that are specific to certain subgroups of patients. On the other hand, we identified WMH and EPVS as early imaging features of SVD that seem to be present in parallel and start progressing together. Their connection has already been reported previously [25]. Still, their clinical relevance surprisingly differed. In line with previous studies that found a strong association of clinical variables with WMH in general and compared to other imaging features [1, 11, 15], we found marked WMH in both clusters with unfavorable stroke outcomes. In line with this finding, of all SVD imaging features, WMH were most strongly associated with stroke outcome in a simple regression. On the other hand, EPVS were not associated with stroke outcome and its removal from the SVD MRI burden score significantly improved the score as a feature for stroke outcome (see supplementary). This is in line with previous studies that found that some SVD features, but not EPVS were associated with stroke outcome and dementia [14, 16]. Hence, the pathological impact of EPVS remains disputable (see also [9]). EPVS might be an early feature that is present before the occurrence of neurological symptoms, therefore being limited as a biomarker. Otherwise, EPVS might be heterogeneous in its presentation, requiring further characterization [26].

A surprising finding in our cluster analysis was that lacunes were common in two very different clusters. The first was the microbleeds SVD cluster with the most pronounced SVD pathology, pronounced microbleeds and worst clinical outcome. The second was a lacunar SVD cluster with favorable clinical outcome and, aside from the largest number of lacunes, medium WMH and EPVS. Accordingly, the number of lacunes was only weakly correlated with post-stroke outcome. Distinct pathologies underlying lacunes could explain this finding and, indeed, previous studies differentiated two types of lacunes. They were identified to be either of a symptomatic type with a rather favorable outcome or a silent type with a less favorable clinical outcome and a high frequency of WMH [27, 28]. This sub-classification of lacunes may well explain our results and holds important implications for the diagnosis and study of SVD. Another study found that the location of lacunes differentiated hypertensive SVD and cerebral amyloid angiopathy in patients with intracerebral hemorrhage [29]. According to the present findings, without further differentiation of subtypes, lacunes appear to be limited as a diagnostic feature of SVD-related stroke outcome.

The high number of microbleeds was the central feature of the most severely affected cluster (microbleeds SVD) and could distinguish this cluster from the clinically more favorable cluster characterized by lacunes. Thus, a large number of microbleeds might offer a biomarker for the unfavorable impact of SVD. This cluster might also include patients with specific or genetic SVD types [30], such as cerebral amyloid angiopathy [31] which usually presents an unfavorable clinical course. Of note, in the present study, no patient suffered intracerebral hemorrhages previous to stroke or within the follow-up period. Nevertheless, the presence of several microbleeds in cluster 5 seems to represent a malignant feature of SVD that is associated with poor stroke outcome.

Previous studies found the SVD MRI burden to provide a generalizable biomarker [13]. However, even given a significant association of the biomarker with stroke outcome, there might be a high potential for refinements. The set of variables to be included and their weighting are important parameters. The present study found EPVS to be unsuited as a prognostic parameter, and especially WMH and microbleeds appeared to be features that differentiated clinical subgroups for stroke outcome. Further, a sub-classification of lacunes might be informative. Importantly, clusters 3 (lacunar SVD) and 4 (pronounced WMH) demonstrated comparable SVD scores, whereas they differed in the pre- and post-stroke level of functional disability. Thus, the value of SVD features in complex prediction algorithms has to be clarified by future studies. Besides, SVD features might be improved by a volumetric or topographic representation [5, 16, 32], which might allow new insights into the typology and progression of SVD.

### Limitations

The subgroups of patients identified in the cluster analysis describe different stages and possibly different progressions of SVD. Since stages of the pathology merge smoothly, the different clusters cannot be interpreted as clearly delineated subgroups. Accordingly, the optimal number of clusters was heterogeneously estimated by different approaches. Therefore, the cluster analysis presented here should be understood as a descriptive approach that allows one to intuitively grasp data patterns.

A larger acute stroke lesion, for example after large vessel occlusion, could mask previous SVD lesions. SVD imaging features can be assessed in the ipsi- and the contralesional brain hemisphere but, still, the current study might have minimally underestimated the SVD burden in some patients with large stroke lesions.

The study investigated SVD only in patients with acute ischemic stroke. Patients with stroke are more likely to be burdened by cardiovascular risk factors. Hence, we believe that SVD features should be less pronounced in populations without stroke. However, the presence of a stroke event likely did not introduce new subtypes of SVD and, with a higher rate of SVD pathology, might even have allowed for better delineation of pathological subgroups.

## Conclusions and perspective

Our study suggests the existence of several distinct pathological processes that are captured by the imaging features for SVD. This underlines the heterogeneity of SVD pathology and the potential necessity of distinct therapeutic and preventive approaches. A refined assessment and representation of existing SVD MRI features are possible next steps to capturing the heterogeneity of SVD pathology and understanding its progression. It remains open to what extent our findings generalize to other samples and other clinical conditions, such as cognitive status, dementia or risk of stroke, and future longitudinal studies are invaluable to this aim.

### Supplementary Information

Below is the link to the electronic supplementary material.

Supplementary file1 (PDF 363 KB)

## Data Availability

Qualified researchers may request access to anonymized patient data, for which proposals need to be approved by the local ethics commission. Commented scripts for MATLAB/R for exact documentation are available at https://doi.org/10.17632/z5pfbjpcd4.1.

## Abbreviations

DWI: Diffusion-weighted imaging
EPVS: Enlarged perivascular spaces
FLAIR: Fluid-attenuated inversion recovery
mRS: Modified Rankin Scale
NIHSS: National Institutes of Health Stroke Scale
SVD: Small vessel disease
WMH: White matter hyperintensities
