# Supplementary material for: A typology of cerebral small vessel disease based on imaging markers
Source: J Neurol. 2023 Jun 27;270(10):4985–94. doi: 10.1007/s00415-023-11831-x (PMC10511610; doi:10.1007/s00415-023-11831-x)
Supplement: Supplementary file 1 — Supplementary file1 (PDF 363 KB) [file 415_2023_11831_MOESM1_ESM.pdf]

# A typology of cerebral small vessel disease based on imaging markers

## Supplemental Online Materials

### Supplementary Table 1: Detailed cluster centroids

Detailed data on the cluster centroids, i.e. average scores across the included variables for each cluster. Data were normalized and partially de-skewed by log transformation before being subjected to the k-means algorithm. Here, data are shown re-transformed into original data space to allow meaningful interpretation. Values are mean (standard deviation). EPVS – enlarged perivascular spaces; WMH PV – periventricular white matter hyperintensities; WMH D – deep white matter hyperintensities.

| Cluster | N Patients | Lacunes | Microbleeds | EPVS | WMH PV | WMH D |
|---|---|---|---|---|---|---|
| 1 *no/mild SVD* | 407 | 0.05 (0.23) | 0.28 (0.76) | 1.35 (0.48) | 0.44 (0.60) | 0.39 (0.49) |
| 2 *intermediate SVD* | 333 | 0.00 (0.05) | 0.32 (0.70) | 2.59 (0.65) | 1.06 (0.57) | 0.95 (0.42) |
| 3 *lacunar SVD* | 173 | 2.08 (1.74) | 0.34 (0.76) | 2.21 (0.82) | 1.17 (0.71) | 1.05 (0.57) |
| 4 *pronounced WMH SVD* | 210 | 0.28 (0.59) | 0.36 (0.66) | 2.33 (0.93) | 2.47 (0.50) | 2.30 (0.57) |
| 5 *microbleeds SVD* | 84 | 1.69 (2.05) | 10.69 (20.99) | 2.68 (0.94) | 2.24 (0.84) | 1.95 (0.87) |
| Total | 1207 | 0.48 (1.18) | 1.04 (6.17) | 2.08 (0.89) | 1.19 (0.97) | 1.08 (0.88) |

### Supplementary Table 2: Detailed statistics for the comparison between clusters

Detailed statistics for the omnibus tests for the comparison of demographic and clinical data between clusters, as shown in Figure 3. mRS – modified Rankin Scale. See Figure 3 for the results of post hoc tests.

| Variable | Statistics | p-value |
|---|---|---|
| Age | F(4,1206) = 93.63 | p < 0.0001 |
| NIHSS 24h | F(4,1152) = 2.73 | p = 0.028 |
| Risk factor score | F(4,1130) = 11.5 | p < 0.0001 |
| Pre-stroke mRS | H(4) = 29.46 | p < 0.0001 |
| mRS 3 months | H(4) = 48.53 | p < 0.0001 |
| SVD MRI burden | H(4) = 734.39 | p < 0.0001 |

## Supplemental analysis – the impact of MRI field strength on SVD typology

We assessed features of SVD by MRI scanners with a field strength of either 1.5T (63.5%) or 3T (36.5%). With varying field strength, however, MRI examination might show different sensitivity in the identification of SVD features. We first evaluated several imaging features as used in the cluster analysis (i.e. with de-skewing of the numbers of lacunes or microbleeds) with t-tests between patients examined with a 1.5T and 3T MRI scanner. Indeed, we found differences in the number of microbleeds ($t(1205)=2.18$; $p < 0.05$), the number of lacunes ($t(1205)=2.20$; $p < 0.05$), and the rating of perivascular spaces ($t(1205)=6.32$; $p < 0.001$) between field strengths.

This finding raised the question of whether the underestimation of certain SVD imaging features by 1.5T MRI could have biased our findings. Hence, we re-ran the k-means cluster analysis with the subsample of 441 patients with 3T MRI. We again searched for 5 clusters and exactly replicated all other factors. The results are shown in Supplementary Figure 1 below. Conceptually, the cluster solution closely resembled the original one in the total sample. Each cluster in the replication could be assigned to one of the original clusters. Differences were mostly minimal and can be explained by the varying sensitivity of 1.5T vs. 3T MRI as well as slightly different cluster borders. In conclusion, any potential underestimation of SVD features should not have affected the main results of our study. Still, magnetic field strength appears to be a factor to consider in studies on imaging markers of SVD, as a small but significant difference in sensitivity between field strengths exists.

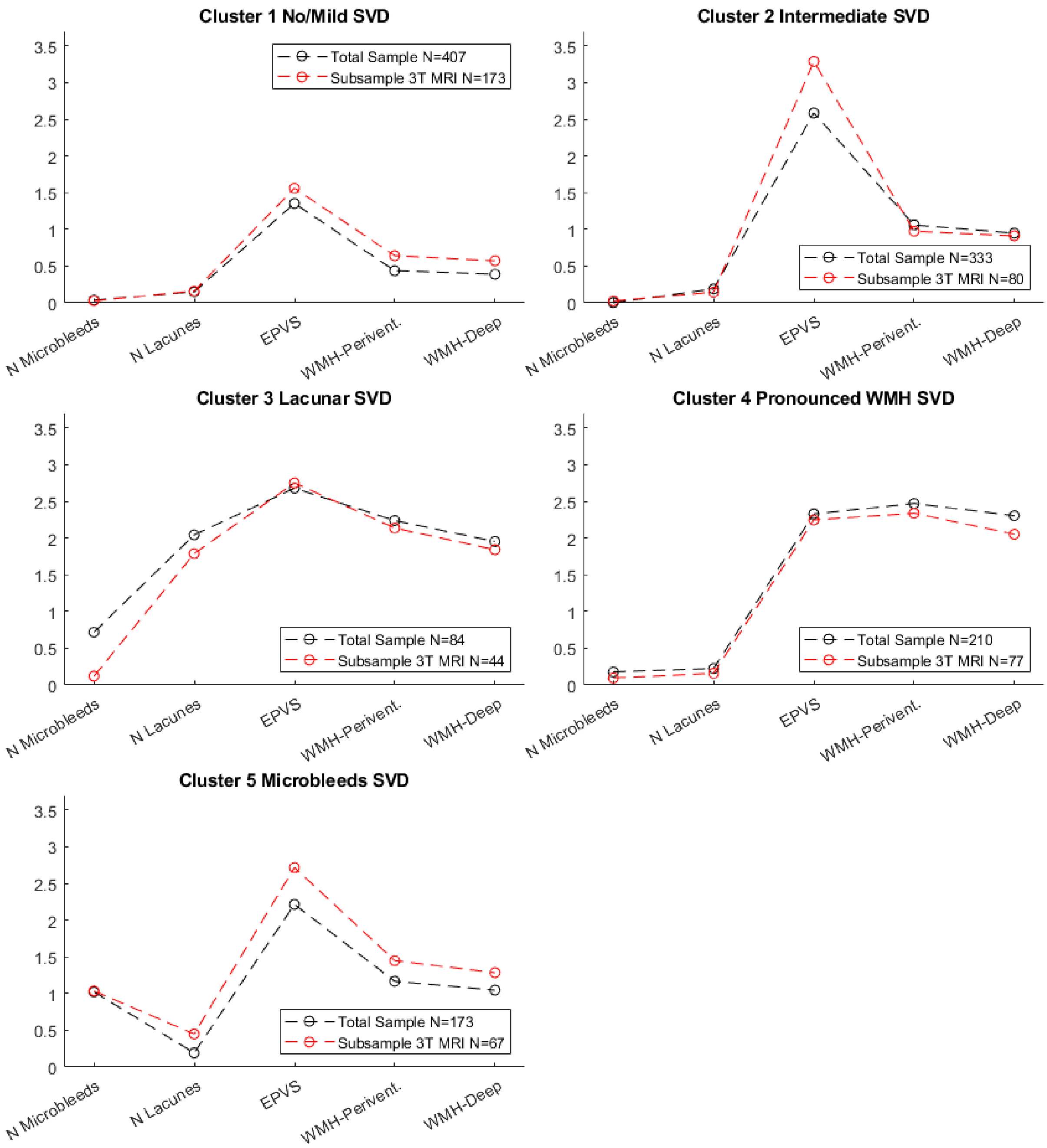


**Supplementary Figure 1: Cluster solution with only 3T MRI**

Results of the replication of the k-means cluster analysis in the subsample of 441 patients assessed with 3T MRI. Values indicate the raw mean values of each feature (i.e. rating/number of) within a cluster. The clusters obtained in the replication are presented with reference to the most similar original clusters of the total sample and with their naming scheme.

## Supplemental analysis – estimation of disease progression from cross-sectional data

In an additional analysis, we aimed to infer the typical disease progression of SVD markers with Bayesian tree models that are capable to represent the progression of binary disease events from cross-sectional data (Beerenwinkel et al., 2005). Studies on the progression of genetic aberrations in tumours (Ketter et al., 2007) and HIV (Beerenwinkel et al., 2005) utilized mutagenetic trees to model disease progression from cross-sectional data. A patient's disease progression within such a model, represented by a so-called genetic progression score, was identified as a biomarker for cancer survival (Rahnenführer et al., 2005). Likewise, models that convert the simple binary assessment of SVD into progression scores might bear the potential to improve biomarkers over simple scorings.

Based on the conditional probabilities between events (i.e. the presence of a type of SVD feature), this technique generates interpretable, directed graph models that visualize the typical order in which SVD features appear. We estimated models with the binary SVD variables for lacunes, microbleeds, EPVS, deep WMH, and periventricular WMH with the Rtreemix package (Bogojeska et al., 2008) in R-software. To account for potentially divergent typical progressions, we allowed for a mixture model of multiple trees (Beerenwinkel et al., 2005) and selected the number of parallel models by evaluation of maximum likelihood. Additionally, we computed for each patient the so-called genetic progression score, a measure that accounts for a patient's disease progression within the estimated tree. The validity of this measure compared to the SVD MRI burden score was assessed by their correlations with stroke outcome, as measured by 3 months post-stroke modified Rankin Scale. Correlations were statistically compared with the R cocor package (Diedenhofen et al., 2015). All statistics were performed at a two-tailed alpha level of $p = 0.05$.

### *Results*

Maximum likelihood evaluation suggested that three tree graph models are sufficient to describe the disease progression underlying the cross-sectional binarized SVD data. As such mixture model includes a star graph to represent a noise component this leaves two interpretable trees (supplementary Figure below). The first tree suggested EPVS as the first disease stage followed by WMH.

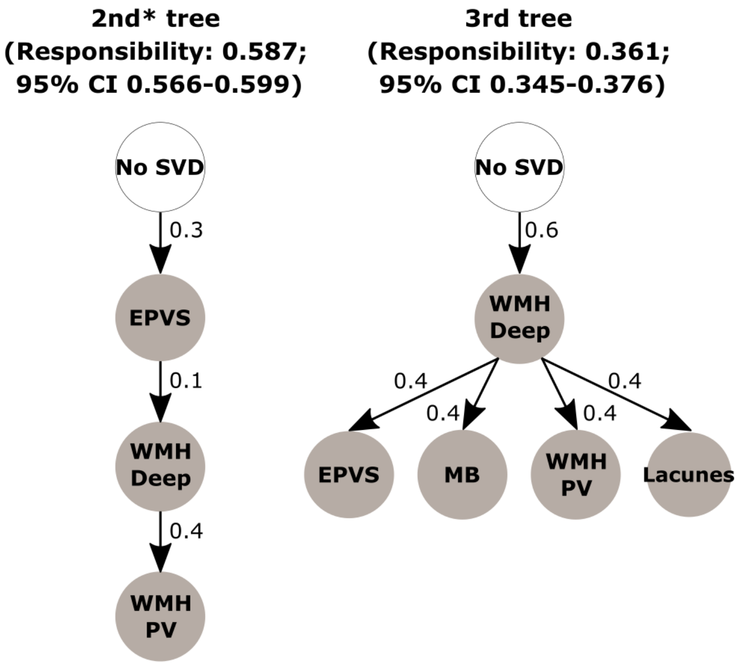


**Supplementary Figure 2: Bayesian tree models for disease progression**

Disease progression as modeled by tree models. Numbers along the edges show the estimated probability to progress from one disease stage to the next. Single vertices emanating from the origin were omitted in the figure. * Visualization starts with the 2nd model, as the first model represents a noise component (responsibility = 0.0521; 95% CI = 0.0516-0.0620; all probabilities = 0.2).

The second tree suggested deep WMH as the first disease state, followed by any of the other SVD pathologies. The progression models were hence to some degree in line with results in the cluster analysis and suggested WMH and EPVS as the most typical early-stage SVD pathology. However, more complex tree models that could explain late-stage SVD pathology were absent. Correspondingly, the Bayesian tree model generated a disease progression score that did not provide an improved biomarker: The SVD MRI burden score and the progression scores carried highly similar information [$r(1205) = 0.950$, $p < 0.001$]. The SVD MRI burden score correlated with stroke outcome by $r(1205) = 0.194$, $p < 0.001$, and the generated SVD progression score correlated with stroke outcome by $r(1205) = 0.191$, $p < 0.001$, which did not significantly differ ($p = .74$). On the other hand, we found that a modified SVD MRI burden score that did not consider EPVS, i.e. a 0 to 3 scale, correlated with stroke outcome by $r(1205) = 0.225$, $p < 0.001$, which outperformed the previous SVD score that included EPVS ($p = 0.013$).

***Discussion***

In line with our interpretation of the cluster analysis, the disease progression models suggested WMH as well as EPVS as typical early MRI signs of SVD. However, they neither modeled late-stage progression paths of SVD nor did the disease progression scores create an improved prognostic biomarker. Hence, our study cannot clarify if the late-stage progression paths of SVD do not follow typical patterns, or if our models with the binary variables were unable to represent pathological processes. Refined or additional SVD features, as well as longitudinal studies, might be required to better understand the progression of SVD pathology.

***Supplementary References***

Beerenwinkel N, Däumer M, Sing T, et al. Estimating HIV Evolutionary Pathways and the Genetic Barrier to Drug Resistance. J. Infect. Dis. 2005;191(11):1953–1960. DOI: 10.1086/430005

Beerenwinkel N, Rahnenführer J, Däumer M, et al. Learning Multiple Evolutionary Pathways from Cross-Sectional Data. J. Comput. Biol. 2005;12(6):584–598. DOI: 10.1089/cmb.2005.12.584

Bogojeska J, Alexa A, Altmann A, et al. Rtreemix: an R package for estimating evolutionary pathways and genetic progression scores.. Bioinformatics 2008;24(20):2391–2. DOI: 10.1093/bioinformatics/btn410

Diedenhofen B, Musch J. cocor: A Comprehensive Solution for the Statistical Comparison of Correlations. PLoS One 2015;10(4):e0121945. DOI: 10.1371/journal.pone.0121945

Ketter R, Urbschat S, Henn W, et al. Application of oncogenetic trees mixtures as a biostatistical model of the clonal cytogenetic evolution of meningiomas. Int. J. Cancer 2007;121(7):1473–1480. DOI: 10.1002/ijc.22855

Rahnenführer J, Beerenwinkel N, Schulz WA, et al. Estimating cancer survival and clinical outcome based on genetic tumor progression scores. Bioinformatics 2005;21(10):2438–2446.
